# Prevalence of Failure due to Adverse Reaction to Metal Debris in Modern, Medium and Large Diameter Metal-on-Metal Hip Replacements – The Effect of Novel Screening Methods: Systematic Review and Metaregression Analysis

**DOI:** 10.1371/journal.pone.0147872

**Published:** 2016-03-01

**Authors:** Aleksi Reito, Olli Lainiala, Petra Elo, Antti Eskelinen

**Affiliations:** Coxa Hospital for Joint Replacement, Biokatu 6b, 33900 Tampere, Finland; Glasgow University, UNITED KINGDOM

## Abstract

Metal-on-metal (MoM) hip replacements were used for almost a decade before adverse reactions to metal debris (ARMD) were found to be a true clinical problem. Currently, there is a paucity of evidence regarding the usefulness of systematic screening for ARMD. We implemented a systematic review and meta-analysis to establish the prevalence of revision confirmed ARMD stratified by the use of different screening protocols in patients with MoM hip replacements. Five levels of screening were identified: no screening (level 0), targeted blood metal ion measurement and/or cross-sectional imaging (level 1), metal ion measurement without imaging (level 2), metal ion measurement with targeted imaging (level 3) and comprehensive screening (both metal ions and imaging for all; level 4). 122 studies meeting our eligibility criteria were included in analysis. These studies included 144 study arms: 100 study arms with hip resurfacings, 33 study arms with large-diameter MoM total hip replacements (THR), and 11 study arms with medium-diameter MoM THRs. For hip resurfacing, the lowest prevalence of ARMD was seen with level 0 screening (pooled prevalence 0.13%) and the highest with level 4 screening (pooled prevalace 9.49%). Pooled prevalence of ARMD with level 0 screening was 0.29% and with level 4 screening 21.3% in the large-diameter MoM THR group. In metaregression analysis of hip resurfacings, level 4 screening was superior with regard to prevalence of ARMD when compared with other levels. In the large diameter THR group level 4 screening was superior to screening 0,2 and 3. These outcomes were irrespective of follow-up time or study publication year. With hip resurfacings, routine cross-sectional imaging regardless of clinical findings is advisable. It is clear, however, that targeted metal ion measurement and/or imaging is not sufficient in the screening for ARMD in any implant concepts. However, economic aspects should be weighed when choosing the preferred screening level.

## Introduction

In the late 1990s, advances in metallurgy and tribology led to a renewed interest in the use of metal-on-metal (MoM) bearings in total hip replacements (THR) [1]. The use of large diameter (LD) femoral heads that mimick the native anatomy of the hip joint requires relatively thin, i.e., 4–8 mm acetabular components to prevent excessive acetabular bone resection. Due to the extreme hardness of the new cobalt-chrome alloy Metasul, it became possible to manufacture these thin acetabular components [1]. With improved fixation techniques, the concept of large-headed femoral components coupled with thin monoblock cups were rapidly adopted for MoM hip resurfacings. Preliminary results with these second generation MoM hip resurfacings were excellent, and the number of hip resurfacings surged in the early 2000s. Later on, LD MoM bearings for cementless stemmed THRs were adopted.

It was only after a decade of use of these contemporary MoM bearings that adverse reaction to metal debris (ARMD) came into focus [2,3]. Metal debris caused by the increased wear of the bearing and/or the corrosion in the neck-head taper in a stemmed MoM THR leads to local soft tissue reactions that include synovitis, necrosis and extra-articular cysts or solid masses, i.e., pseudotumours [4–8]. ARMD is an umbrella term proposed by Langton et al. in 2010 to describe all the microscopic and macroscopic findings **t**hat were seen in revision surgeries performed on patients with MoM hip replacements who suffered from unexplained pain [2].

Nowadays, revision surgery is considered if a large thick-walled pseudotumour is seen in MRI, or if extremely high metal ion levels (>10–20 ppb) are found in the serum or whole blood (WB). Prior to 2010, MoM hip resurfacings and modular MoM THRs produced excellent results in young and active patients. Since 2010, however, there has been a drastic decline in the use of MoM hip replacements due to the higher than anticipated prevalence of ARMD and subsequent high failure rates [9,10].

An extensive amount of research has been published on ARMD. Novel screening methods such as blood metal ion measurement and cross-sectional imaging have offered the profound possibility to investigate the etiopathogenesis, clinical history and clinical manifestation of ARMD [2,4,5,11–14]. Both blood metal ion measurement and cross-sectional imaging have been proven to be useful in the diagnostics of ARMD [4,5,15]. However, there is a paucity of current literature regarding the usefulness of systematic screening for ARMD. Current guidelines on how to follow-up patients with MoM hip replacements lack sufficient evidence [16–18]. The intensity and coverage of such screening is strongly associated with the costs related to the surveillance of patients with MoM hip replacements.

Although the number of patients receiving MoM THRs has decreased drastically in recent years,. there are still a vast number of patients with MoM hip replacement still *in situ*. Thus, there is a clear need for sufficient evidence on how to manage patients with MoM hip replacements.

The aim of this study was to conduct a systematic review and meta-analysis of current literature to establish the prevalence of revision confirmed ARMD stratified by the use of different screening protocols. A second aim was to explore the possible confounding effects of follow-up time and year of publication of the study on the prevalence of revision confirmed ARMD by performing a meta-regression analysis.

## Methods

### Eligibility criteria

A study was deemed eligible for our analysis if 1) it included an original patient cohort operated on with a single disclosed implant, 2) the implant used in the study was MoM hip resurfacing or MoM THR with a femoral head diameter of 36 mm or larger, and 3) the reasons for the revisions were clearly stated or the operative findings in the revision surgery were outlined. An original patient cohort means a clearly defined population of patients operated on with a certain implant within a certain time interval at disclosed hospital(s). A study was excluded if more than one implant was used and the number of patients for each implant was not given. Moreover, a study was excluded if 1) it included patients referred from elsewhere to the place where the study was carried out (violation of eligibility criteria 1), 2) more than one implant was used, but revisions were not stratified according to the implant used, 3) the reasons for all revisions were not given, 4) the size of a study arm for a single implant was less than 20 hips.

Furthermore, if a study included a study arm or a subcohort of a study arm, which had been included in a previous study and in both studies a similar screening method was used, the more recent study was included in our analysis. However, if the two studies included an identical study arm or a subcohort of a study arm but the more recent study implemented a different level of screening, both studies were included in our analyses since the primary aim of our study was to investigate the effect of screening levels on the prevalence of revision confirmed ARMD.

### Information sources and search strategy

The review was done according to PRISMA checklist (S1 Checklist). We developed a search strategy that was implemented in the PubMed and Scopus databases. Since our objective was to establish a pooled prevalence of ARMD seen with contemporary MoM hip replacements, we started our search of these databases from the year 1995. The search was conducted in May 2015.

We performed two searches of these two databases. The following search strategy was used first: “(metal-on-metal AND hip) OR (("hip resurfacing" OR ("surface replacement" AND hip) OR "large-diameter total hip") NOT (metal-on-metal))”. A second search was carried out afterwards that was combined with the first search using the Boolean operator NOT in order to remove duplicates: “((recap AND hip) OR (magnum AND hip) OR (cormet AND hip) OR (durom AND hip) OR (conserve AND hip) OR (pinnacle AND hip) OR (asr AND hip) OR (m2a AND hip) OR (birmingham AND hip) OR (mitch AND hip) OR (adept AND hip))”. The latter search phrase outlines the most commonly used MoM hip replacement brand names.

### Study selection

All the records retrieved from the two databases using our search strategy were screened. An assessment of duplicate references was not performed. Abstracts of all the records were assessed. Studies that outlined the use of any “metal-on-metal” hip implant or a hip implant by a brand name along with any clinical outcome (patient reported outcome score, survival rate, failure rate, complication rate, revision rate, deaths, levels of metal ion levels, cross-sectional imaging findings) were selected for eligibility assessment. All the studies meeting our eligibility criteria were selected for both systematic review and meta-analysis that was conducted using the metaregression technique.

### Data extraction

The number of hips operated on with each given implant was recorded. Implant concept and publication year of the study were also recorded. Three categories for implant concept were used: 1) hip resurfacings or surface arthroplasties, 2) LD MoM THRs (femoral diameter >44 mm), which comprise identical bearing systems as in hip resurfacings coupled with a mainly cementless stem, and 3) MoM THR with a femoral diameter of 44 mm or smaller [medium-diameter (MD) MoM THR]. The last group included two fixed-sized MoM THRs: the Pinnacle 36 mm MoM THR and the M2a-38 MoM THR with a 38 mm femoral diameter. Small-diameter (<36 mm) MoM hip replacements were not included in the present study. Two studies included patients operated on with a Birmingham Mid-Head Resection (BMHR) device. These were included in the hip-resurfacing group due to their similarity. Follow-up time was recorded. We did not differentiate between mean and median follow-up times. The use of metal ion level measurement in serum or WB was assessed as well as the use of any cross-sectional imaging modality. In short, five levels of screening were identified: no screening (level 0), targeted blood metal ion measurement and/or cross-sectional imaging (level 1), blood metal ion measurement without imaging (level 2), blood metal ion measurement with targeted imaging (level 3) and comprehensive screening (both blood metal ions and imaging for all; level 4). If neither screening method was used, the level of screening was labeled “None” (Level 0). If prerevision details that included metal ion levels and/or imaging findings were described in single patients in the results section, the use of screening was considered lacking unless there was a protocol rationale in methods section that described the use of these screening methods, i.e., patients with a complaint underwent an MRI scan. If blood metal ion measurements were performed in a subset of patients and no cross-sectional imaging was performed or if cross-sectional imaging was performed in a subset of patients without any given metal ion data, the level of screening was labeled “Targeted CoCr and/or imaging” (Level 1). Again, if prerevision imaging findings or metal ion levels were described in single patients without any protocol rationale detailed in the methods section, these screening methods were considered to be lacking. If all patients underwent a metal ion measurement without any imaging protocol outlined in the methods section, the level of screening was labeled “CoCr without imaging” (Level 2). If targeted imaging was used along with a routine (full coverage) metal ion measurement, the level of screening was labeled “CoCr with targeted imaging” (Level 3). If all patients underwent both metal ion measurement and cross-sectional imaging, the level of screening was labeled “Comprehensive” (Level 4). The modality of the imaging was recorded. We did not differentiate between serum and WB measurements.

The number and reasons for the revisions were recorded. Detailed prerevision and perioperative findings were assessed if described. The following reasons for the revision were considered to be ARMD: “ARM(e)D”, “Adverse wear”,”adverse local tissue reaction (ALTR)”,”adverse tissue reaction (ATR)”, “metallosis”, “pseudotumour” and “synovitis”. “Elevated metal ion levels” or “component/cup malposition” as reasons for revision were not considered to be ARMD unless perioperative findings were described. The definition of ARMD was also met if perioperative findings were described, that is if the operative findings described included the terms “metallosis”, “synovitis”, “pseudotumour”, “necrotic substance/tissue” and the case(s) outlined were considered to have failed due to ARMD. Cases with ARMD as revision indication but that also included a clear statement about component loosening were not included in our analyses.

### Summary measures

The primary summary measure was the prevalence of ARMD. This was calculated by dividing the total number of revisions due to ARMD by the total number of hips included in the study. Confounding variables included in the meta-regression were follow-up time, year of publication and level of screening. This information was extracted as described in the previous section.

### Data synthesis

The Q-statistic was used to assess heterogeneity across the studies. If the Q-statistic suggested high heterogeneity (p-value< 0.1), a random effects model was used instead of a fixed effects model. The amount of heterogeneity was assessed using the I^2^-measure. The DerSimonian-Laird estimator was used as a random effects model when needed. Arcsine transformation was used for the summary measure (prevalence o revision confirmed ARMD). We preferred this to logit transformation because zero prevalence was overrepresented in our study. With logit transformation we would have been obliged to choose a random increment to be added to the zero summary measures. We would not have had any reasonable value for this. Metaregression analysis was used to assess whether differences in the prevalence of revision confirmed ARMD across different levels of screening remained after adjusting for the year of publication and follow-up time. All analyses were stratified by the implant concept (HR/LD THR/MD THR). Finally, we carried out a “best case” sensitivity analysis by performing all the aforementioned analyses using only study arms with patients operated on with Birmingham Hip Resurfacing (BHR, Smith&Nephew, Warwick, United Kingdom).

## Results

**Fig 1:**
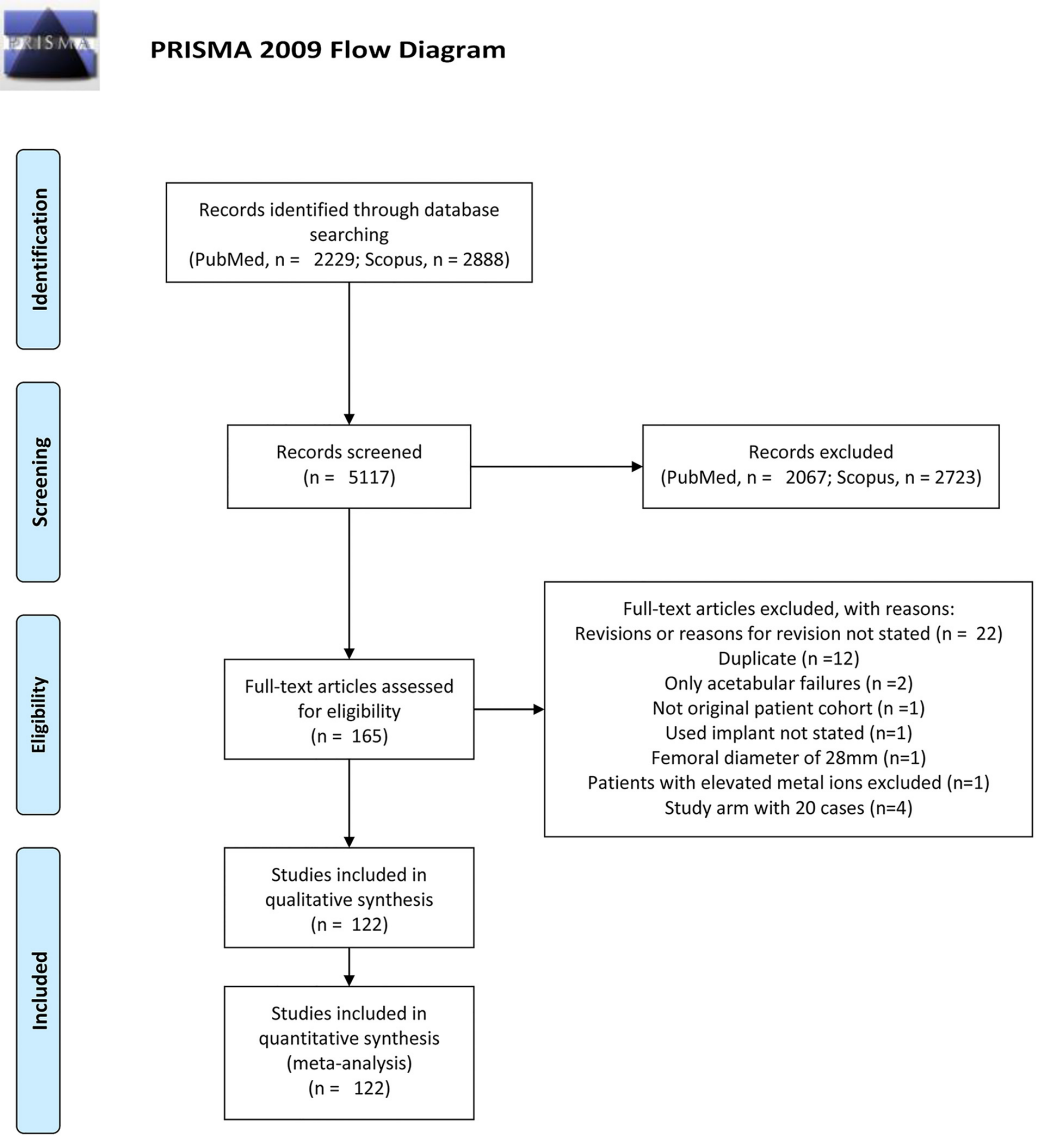
Flow chart of the study selection.

A PRISMA flow diagram of the study selection process is shown in Fig 1. In total, 122 studies were included. These studies comprised 145 study arms (Table 1) (S1 Datafile). The median number of hips in the study arms was 128 (range 20–3095). The most commonly used implant was BHR (Table 2). Patients in 48 study arms were operated on with BHR. In total, 100 study arms included patients operated on with a hip-resurfacing device [14,15,19–99]. Thirty-three study arms included patients with LD-THA [35,56,79,95,100–121]. The most commonly used LD THA was the articular surface replacement (ASR) XL THR (DePuy Orthopedics, Warsaw, IN, USA). In general, the stems used with LD MoM bearing couples varied greatly. MD THR was used in 11 study arms [94,112,120,122–129]. In most of the studies, a traditional or conventional follow-up protocol without metal ion measurement and cross-sectional imaging was used (Table 2). Distribution of the follow-up time and year of publication are presented in Table 2.

**Table 1:** Details of the study arms. Within implant concept, study arms are primarily ordered by brand name, secondarily by screening level.

Study	Patients	Hips	Acetabular side	Femoral side	Metal ion measurement	Targeted measurement performed	Cross-sectional imaging	Targeted imaging	Imaging modality	Level of screening	Follow-up in years	Revisions due to ARMD	Prevalence of ARMD
Hip resurfacings													
Bergeron et al. 2009 [25]	209	228	ASR	ASR	No		No		No	0	2.9	0	0.000
Jameson et al. [51]	192	214	ASR	ASR	No		No		No	0	3.6	6	0.028
Malhotra et al. 2012 [60]	23	32	ASR	ASR	No		No		No	0	3.6	0	0.000
Siebel et al. 2006	300	300	ASR	ASR	No		No		No	0	0.56	0	0
Whitehouse et al. 2013 [94]	0	76	ASR	ASR	No		No		No	0	4.9	4	0.053
Kadar et al. 2013 [53]	125	139	ASR	ASR	Target	Symptoms, suboptimal cup position, small femoral size, surgeon concern	Target	Symptoms, suboptimal cup position, small femoral size, surgeon concern	MRI	1	3.5	1	0.007
Whitwell et al. 2012 (Arm 1) [95]	0	21	ASR	ASR	Target	Patients with symptoms	Target	Patients with symptoms	US	1	5.2	3	0.143
Isaac et al. 2009 [136]	0	77	ASR	ASR	All		No		No	2	2.0	0	0.000
Langton 2011a (Arm 1) [14]	0	59	ASR	ASR	All		No		No	2	2.6	2	0.034
Langton 2011a (Arm 4) [14]	0	430	ASR	ASR	All		No		No	2	3.1	27	0.063
Shemesh et al. 2014 [83]	0	49	ASR	ASR	All		No		No	2	4.1	0	0.000
Fernandez et al. 2014 (Arm 1) [35]	0	60	ASR	ASR	All		Target		CT	3	2.9	2	0.033
Reito et al. 2013 (Arm 1) [79]	142	168	ASR	ASR	All		All		MRI	4	5.3	42	0.250
Aulakh et al. 2010 (Arm 1) [21]	95	101	BHR	BHR	No		No		No	0	3.0	0	0.000
Aulakh et al. 2010 (Arm 2) [21]	97	101	BHR	BHR	No		No		No	0	2.0	0	0.000
Azam et al. 2015 [22]	222	224	BHR	BHR	No		No		No	0	12.1	4	0.018
Baker et al. 2011 [23]	0	63	BHR	BHR	No		No		No	0	9.0	0	0.000
Bose et al. 2010 [28]	71	96	BHR	BHR	No		No		No	0	5.4	0	0.000
Coulter et al. 2012 [29]	213	230	BHR	BHR	No		No		No	0	10.4	3	0.013
De Smet 2005 [32]	0	252	BHR	BHR	No		No		No	0	2.8	0	0.000
Della Valle et al. 2009 [33]	0	537	BHR	BHR	No		No		No	0	0.9	0	0.000
Delport et al. 2011 (Arm 1) [34]	28	28	BHR	BHR	No		No		No	0	4.8	0	0.000
Fink Barnes et al. 2014 [36]	80	89	BHR	BHR	No		No		No	0	3.6	0	0.000
Giannini et al. 2011 [38]	134	142	BHR	BHR	No		No		No	0	6.1	0	0.000
El Hachmi et al. 2014 [43]	141	151	BHR	BHR	No		No		No	0	4.2	0	0.000
Heilpern et al. 2008 [46]	98	110	BHR	BHR	No		No		No	0	5.9	0	0.000
Khan et al. 2009 [54]	653	679	BHR	BHR	No		No		No	0	6.0	1	0.001
Madhu et al. 2011 [59]	104	120	BHR	BHR	No		No		No	0	7.0	0	0.000
Marulanda et al. 2008 [137]	0	230	BHR	BHR	No		No		No	0	1.30	0	0
McAndrew et al. 2007 [62]	155	180	BHR	BHR	No		No		No	0	2.6	0	0.000
McBryde et al. 2010 [63]	1826	2123	BHR	BHR	No		No		No	0	3.5	0	0.000
McMinn et al. 2011b [65]	0	3095	BHR	BHR	No		No		No	0	8.0	10	0.003
Naal et al. 2009 [138]	0	22	BHR	BHR	No		No		No	0	3.6	0	0.000
Ollivere et al. 2008 [71]	0	463	BHR	BHR	No		No		No	0	3.6	7	0.015
Pailhe et al. 2013 (Arm 2) [72]	0	42	BHR	BHR	No		No		No	0	3.2	0	0.000
Patel et al. 2014 [74]	85	109	BHR	BHR	No		No		No	0	5.2	0	0.000
Rahman et al. 2010 [77]	302	329	BHR	BHR	No		No		No	0	5.0	1	0.003
Reito et al. 2011 [78]	126	144	BHR	BHR	No		No		No	0	6.0	2	0.014
Sandiford et al. 2014 [82]	107	109	BHR	BHR	No		No		No	0	8.2	3	0.028
Swank et al. 2009 [87]	0	128	BHR	BHR	No		No		No	0	2.0	0	0.000
Takamura et al. 2014 (Arm 1) [88]	0	115	BHR	BHR	No		No		No	0	2.8	2	0.017
Takamura et al. 2014 (Arm 2) [88]	0	236	BHR	BHR	No		No		No	0	2.2	1	0.004
Whitehouse et al. 2013 [139]	0	103	BHR	BHR	No		No		No	0	4.3	4	0.039
Witzleb et al. 2008	263	300	BHR	BHR	No		No		No	0	2.00	0	0
Murray et al. 2012 [66]	554	646	BHR	BHR	No		Target	OHS<33	MRI	1	8.0	26	0.040
Holland et al. 2012 [47]	90	100	BHR	BHR	Target		No		No	1	9.6	2	0.020
Kostensalo et al. 2014 (Arm 1) [56]	0	249	BHR	BHR	Target	ns	No		No	1	6.2	3	0.012
Nam et al. 2012 (Arm 2) [69]	126	137	BHR	BHR	Target	ns	No		No	1	5.7	2	0.015
Pailhe et al. 2014 [73]	162	180	BHR	BHR	Target	Patient with symptoms	No		No	1	6.0	1	0.006
Daniel et al. 2014 [31]	886	1000	BHR	BHR	Target	Patients with longest follow-up	Target	Patients with longest follow-up	CT	1	13.7	7	0.007
Langton 2011a (Arm 2) [14]	0	1922	BHR	BHR	All		No		No	2	5.7	23	0.012
Langton 2011a (Arm 5) [14]	0	180	BHR	BHR	All		No		No	2	5.4	1	0.006
Langton 2011a (Arm 6) [14]	0	674	BHR	BHR	All		No		No	2	5.4	3	0.004
Robinson et al. 2014 (Arm 1) [81]	0	120	BHR	BHR	All		No		No	2	4.2	1	0.008
Su et al. 2014 [86]	265	293	BHR	BHR	All		No		No	2	3.6	1	0.003
van der Straeten et al. 2013 [89]	232	250	BHR	BHR	All		No		No	2	10.8	4	0.016
Hartmann et al. 2012 [45]	95	100	BHR	BHR	All		Target	Co or Cr level >10 ppb	CT	3	10.0	3	0.030
Reito et al. 2014 [15]	219	261	BHR	BHR	All		Target	Patients with symptoms, Co or Cr level >5 ppb	US/MRI	3	10.4	11	0.042
Bisschop et al. 2013 [26]	129	149	BHR	BHR	All		All		CT	4	3.4	8	0.054
Haddad et al. 2015	40	47	BHR	BHR	All		All		MRI	1	12.1	0	0.000
Junnila et al. 2015 [52]	32	42	BHR	BHR	All		All		MRI	4	6.7	4	0.095
Radtke et al. 2014 [75]	75	85	Bionic	Bionic	No		No		No	0	5.0	0	0.000
McMinn et al. 2011a [140]	164	171	BMHR	BMHR	No		No		No	0	3.5	0	0.000
Rahman et al. 2011 [76]	34	35	BMHR	BMHR	No		No		No	0	2.8	0	0.000
Amstutz et al. 2011	0	1107	Conserve+	Conserve+	No		No		No	0	6.8	3	0.003
Arndt et al. 2013 [20]	0	55	Conserve+	Conserve+	No		No		No	0	4.2	0	0.000
Kim et al. 2008 [24]	0	200	Conserve+	Conserve+	No		No		No	0	2.6	0	0.000
Fowble et al. 2009 [141]	50	50	Conserve+	Conserve+	No		No		No	0	3.2	0	0.000
Glyn-Jones et al. 2009 (Arm 1) [39]	0	606	Conserve+	Conserve+	No		No		No	0	4.0	8	0.013
Marker et al. 2010 [61]	0	361	Conserve+	Conserve+	No		No		No	0	4.9	0	0.000
McGrath et al. 2008 (Arm 1) [64]	0	42	Conserve+	Conserve+	No		No		No	0	3.0	0	0.000
McGrath et al. 2008 (Arm 2) [64]	0	153	Conserve+	Conserve+	No		No		No	0	3.0	0	0.000
Newman et al. 2008 [70]	120	126	Conserve+	Conserve+	No		No		No	0	1.0	0	0.000
Ribas et al. 2014 [80]	450	486	Conserve+	Conserve+	No		No		No	0	7.2	1	0.002
Wang et al. 2012 [80]	34	37	Conserve+	Conserve+	No		No		No	0	5.9	0	0.000
Woon et al. 2013 [97]	46	53	Conserve+	Conserve+	No		No		No	0	8.3	0	0.000
Zylberberg et al. 2015 [99]	458	548	Conserve+	Conserve+	No		No		No	0	6.6	4	0.007
Nam et al. 2012 (Arm 1) [69]	124	137	Conserve+	Conserve+	Target	ns	No		No	1	5.5	1	0.007
Bisseling et al. 2015 [27]	32	38	Conserve+	Conserve+	All		No		No	2	4.8	2	0.053
Kim et al. 2011 [142]	97	97	Conserve+	Conserve+	All		No		No	2	2.0	0	0.000
Langton 2011a (Arm 3) [14]	0	961	Conserve+	Conserve+	All		No		No	2	2.8	4	0.004
Yang et al. 2011 [98]	25	25	Conserve+	Conserve+	All		No		No	2	2.0	0	0.000
Stulberg et al. 2008 [85]	0	337	Cormet Hybrid	Cormet Hybrid	No		No		No	0	2.0	0	0.000
Gross et al. 2012 [40]	329	373	Cormet Uncemented	Cormet Cemented	No		No		No	0	8.0	2	0.005
Issa et al. 2013 [50]	114	120	Cormet Uncemented	Cormet Cemented	No		No		No	0	3.5	0	0.000
Kordas et al. 2012	215	234	Cormet Uncemented	Cormet Cemented	No		No		No	0	5.0	0	0.000
Madadi et al. 2011 [58]	0	52	Cormet Uncemented	Cormet Cemented	No		No		No	0	3.4	0	0.000
Gross et al. 2008 [42]	18	20	Cormet Uncemented	Cormet Uncemented	No		No		No	0	7.4	0	0.000
Hull et al. 2011 [48]	131	135	Cormet Uncemented	Cormet Uncemented	No		No		No	0	2.9	0	0.000
Leclerq et al. 2013 [57]	580	644	DUROM	DUROM	No		No		No	0	2.8	1	0.002
Naal et al. 2011 [67]	91	100	DUROM	DUROM	No		No		No	0	5.0	0	0.000
Pailhe et al. 2013 (Arm 1) [72]	0	100	DUROM	DUROM	No		No		No	0	3.2	0	0.000
Vendittoli et al. 2013 [93]	0	112	DUROM	DUROM	Target	Patients with follow-up of >5 years	No		No	1	8.0	1	0.009
Robinson et al. 2014 (Arm 2) [143]	0	240	DUROM	DUROM	All		No		No	2	4.6	0	0.000
van der Weegen et al. 2012 [91]	240	280	M2a-Magnum	ReCap	No		Target	Patients with symptoms	US	1	3.3	0	0.000
van der Weegen et al. 2014 [90]	235	271	M2a-Magnum	ReCap	All		All		MRI	4	4.6	9	0.033
Delport et al. 2011 (Arm 2) [34]	28	28	M2a-Magnum	ReCap	No		No		No	0	1.4	0	0.000
Glyn-Jones et al. 2009 (Arm 2) [39]	0	128	M2a-Magnum	ReCap	No		No		No	0	4.0	0	0.000
Gross et al. 2013 [41]	0	2166	M2a-Magnum	ReCap	Target		Target		CT/MRI	1	4.0	5	0.002
Daniel et al. 2010 [30]	160	184	McMinn	McMinn	No		No		No	0	10.6	0	0.000
LD THR													
Bolland et al. 2011 [102]	185	199	Adept/BHR	CPT	All		No		No	2	5.2	14	0.070
Steele et al. 2008 [119]	109	120	ASR XL	ns	No		No		No	0	1.6	4	0.033
Wynn-Jones et al. 2011 [121]	54	62	ASR XL	Corail	No		All		MRI	1	2.5	7	0.113
Whitwell et al. 2012 (Arm 2) [95]	0	100	ASR XL	Corail	Target	Patients with symptoms	Target	Patients with symptoms	US	1	4.4	17	0.170
Hug et al. 2013 [108]	0	149	ASR XL	Summit	Target	Symptoms or patient willingness	Target	Patients scheduled for revision with elevated metal ion levels and suspicion for ARMD	MRI	1	3.3	24	0.161
Langton 2011b [110]	0	87	ASR XL	57 S-ROM, 30 Corail	All		No		No	2	4.0	25	0.287
Lavigne et al. 2011 (Arm 3) [113]	0	32	ASR XL	Trilock	All		No		No	2	2.0	0	0.000
Fernandez et al. 2014 (Arm 2) [35]	0	23	ASR XL	Proxima	All		Target	ns	CT	3	2.9	0	0.000
Reito et al. 2015 [117]	196	225	ASR XL	149 Summit, 53 Corail, 21 S-ROM, 1 Proxima	All		All		MRI	4	5.4	73	0.324
Reito et al. 2013 (Arm 2) [79]	281	312	ASR XL	233 Summit, 54 Corail, 24 S-ROM, 1 Proxima	All		All		MRI	4	4.6	96	0.308
Cip et al. 2014 [144]	88	99	ASR XL	97 CoxaFit, 2 ARGE Geradschaft	All		All		CT	4	3.5	26	0.263
Kostensalo et al. 2014 (Arm 2) [56]	0	39	BHR	Synergy	Target	ns	No		No	1	3.9	0	0.000
Hosny et al. 2013 [107]	41	44	BHR	Synergy	Target	"Some patients"	Target	Patient with symptoms or elevated metal ion level (no cut-off stated)	MRI	1	5.0	2	0.045
Lavigne et al. 2011 (Arm 4) [113]	0	29	BHR	Anthology	All		No		No	2	2.0	0	0.000
Chatrath et al. 2013 [104]	88	89	Conserve+	Profemur	No		No		No	0	2.5	3	0.034
Levy et al. 2013 [114]	0	66	Conserve+	Profemur	Target	"Patients with suspected failing hips"	Target	"Patients with suspected failing hips"	CT/US	1	1.3	7	0.106
Lardanchet et al. 2012 (Arm 3) [111]	0	20	Conserve+	16 Profemur L, 4 Contact Evolution	All		No		No	2	1.0	0	0.000
Hasegawa et al. 2013 [106]	98	108	Cormet	Cti II	All		All		MRI	4	3.8	5	0.046
Mertl et al. 2010 [115]	107	111	DUROM	78 Muller type, 28 Emeraude	No		No		No	0	2.5	0	0.000
Berton et al. 2010 [101]	92	100	DUROM	Zweymuller SL	No		No		No	0	4.8	1	0.010
Saragaglia et al. 2015 [118]	165	177	DUROM	PF Stem	No		Target	Unexplained pain, osteolysis, bone cysts	US	1	6.7	4	0.023
Lavigne et al. 2011 (Arm 1) [113]	0	42	DUROM	CLS Spotorno	All		No		No	2	2.0	1	0.024
Lardanchet et al. 2012 (Arm 1) [111]	0	24	DUROMTHA	20 Contact Evolution, 4 Profemur L	All		No		No	2	1.0	2	0.083
Bayley et al. 2015 [100]	215	258	M2a-Magnum	Mallory Head	All		Target	Follow-up >1 year	US	3	4.5	0	0.000
Kostensalo et al. 2012 [109]	635	691	M2a-Magnum/ReCap	Bimetric	No		No		No	0	1.0	0	0.000
Latteier et al. 2011 (Arm 2) [112]	0	487	M2a-Magnum/ReCap	ns	No		No		No	0	5.0	3	0.006
Lardanchet et al. 2012 (Arm 2) [111]	0	23	M2a-Magnum/ReCap	Exception	All		No		No	2	1.0	0	0.000
Lavigne et al. 2011 (Arm 2) [113]	0	34	M2a-Magnum/ReCap	Taperloc	All		No		No	2	2.0	0	0.000
Sturup et al. 2012 (Arm 2) [120]	0	271	M2a-Magnum/ReCap	ns	All		Target	Patients with symptoms	CT	3	3.5	1	0.004
Bosker et al. 2012 [103]	119	120	M2a-Magnum/ReCap	Bimetric	All		All		CT	4	3.6	13	0.108
Mokka et al. 2013 [116]	74	80	M2a-Magnum/ReCap	BiMetric	All		All		MRI	4	6.0	3	0.038
Kostensalo et al. 2014 (Arm 3) [56]	0	41	R3	Synergy	Target	ns	No		No	1	2.3	2	0.049
Dramis et al. 2014 [105]	46	50	R3	30 Anthology, 18 CPCS, 2 SL Plus	All		Target	Symptomatic patients and those with "adverse investigations"	MRI	3	3.8	12	0.240
MD THR													
Latteier et al. 2011 (Arm 1) [112]	0	750	M2a-38	ns	No		No		No	0	5.0	11	0.015
Sturup et al. 2012 (Arm 1) [120]	0	85	M2a-38	ns	All		Target	Patients with symptoms	CT	3	3.5	0	0.000
Smeekes et al. 2015 [129]	351	377	M2a-38	Taperloc	All		Target	Patients with symptoms, Co or Cr level >5 ppb	MRI	3	2.5	51	0.135
Barret et al. 2012 [122]	0	779	Pinnacle	310 Summit Porocoat, 234 S-ROM, 139 Summit Duofix, 47 Prodigy, 35 AML, 11 Summit Cemented, 2 Replica, 1 Endurance	No		No		No	0	4.2	8	0.010
Kindsfater et al. 2012 [125]	0	95	Pinnacle	86 S-ROM, 8 Summit Cemented, 1 Summit	No		No		No	0	6.0	0	0.000
Engh et al. 2010 [124]	126	131	Pinnacle	AML/Prodigy (numbers ns)	No		No		No	0	5.6	0	0.000
Whitehouse et al. 2013 [94]	0	99	Pinnacle	Corail	No		No		No	0	4.0	3	0.030
Bernasek et al. 2013 [123]	0	354	Pinnacle	Summit	No		No		No	0	6.8	0	0.000
Schouten et al. 2012 [128]	0	34	Pinnacle	Corail	All		No		No	2	1.0	0	0.000
Matharu et al. 2014 [127]	511	578	Pinnacle	Corail	All		Target	Co or Cr level >7 ppb	MRI/US	3	5.0	17	0.029
Lainiala et al. 2014 [126]	430	371	Pinnacle System	398 Summit, 17 Corail, 14 S-ROM, 1 Prodigy	All		Target	Patients with symptoms, Co or Cr level >5 ppb	MRI	3	7.5	32	0.086

**Table 2:** Summary data of the study arms.

**Implant used**			**Study arms**
	Hip resurfacing (HR)		
		BHR	48
		Conserve+	18
		ASR	13
		DUROM	5
		Cormet	5
		Recap—M2a-Magnum	5
		Birmingham Mid Head Resection	2
		Cormet Uncemented	2
		Bionic	1
		McMinn	1
	Large-diameter total hip replacements (LD-THA)		
		ASR XL–Mixed stems	10
		DUROM–Mixed stems	5
		ReCap-M2a-Magnum—Mixed stems	8
		BHR–Mixed stems	3
		R3 –Mixed stems	2
		Conserve+—Mixed stems	2
		Adept/BHR—CPT	1
		Cormet–Cti II	1
		Adept—CPT	1
	Medium-diameter total hip replacement (MD-THA)		
		Pinnacle–Mixed stems	8
		M2a-38 –Mixed stems	3
**Screening method**			**Study arms**
	Level 0		
		None	76
	Level 1		
		Targeted blood metal ions without imaging	8
		Targeted imaging without blood metal ions	3
		Targeted blood metal ions with targeted imaging	9
	Level 2		
		Blood metal ions without imaging	26
		Cross-sectional imaging without blood metal ions	1
	Level 3		
		Blood metal ions with targeted imaging	11
	Level 4		
		Blood metal ions AND cross-sectional imaging	10
**Year of publication**			**Study arms**
		2005	1
		2006	1
		2007	1
		2008	8
		2009	12
		2010	11
		2011	31
		2012	23
		2013	23
		2014	24
		2015	9
**Follow-up time**			**Study arms**
		≤2 years	23
		2–4 years	51
		4–6 years	43
		6–8 years	15
		>8 years	12

**Fig 2:**
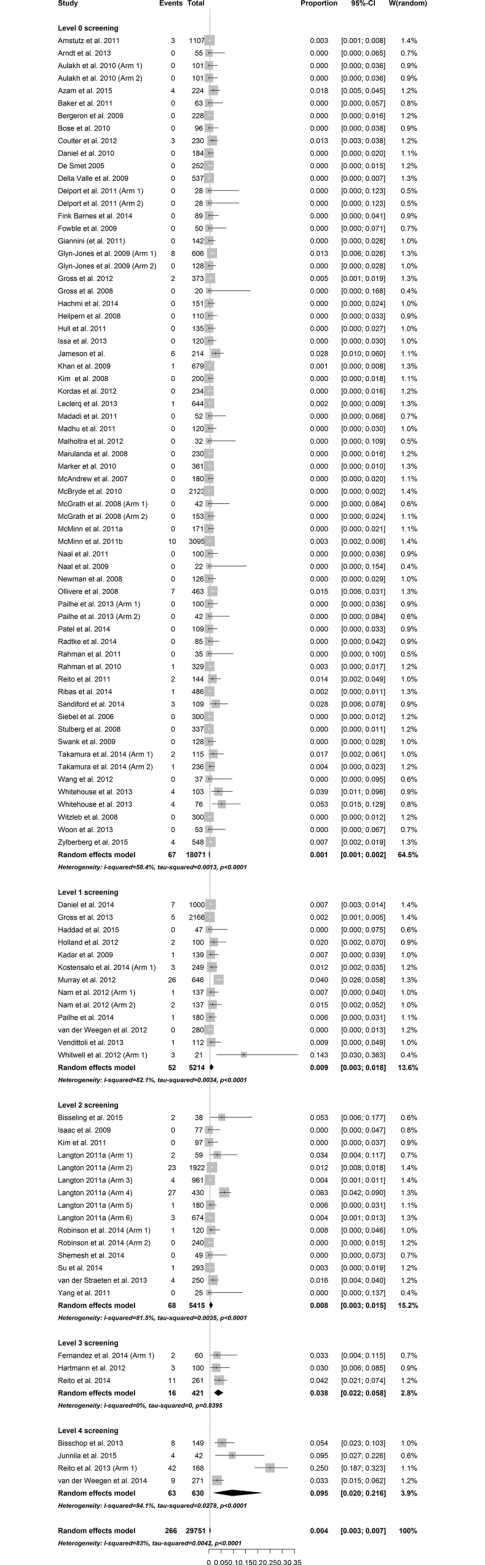
Forest plot of prevalence of ARMD in the HR group stratified by level of screening.

**Fig 3:**
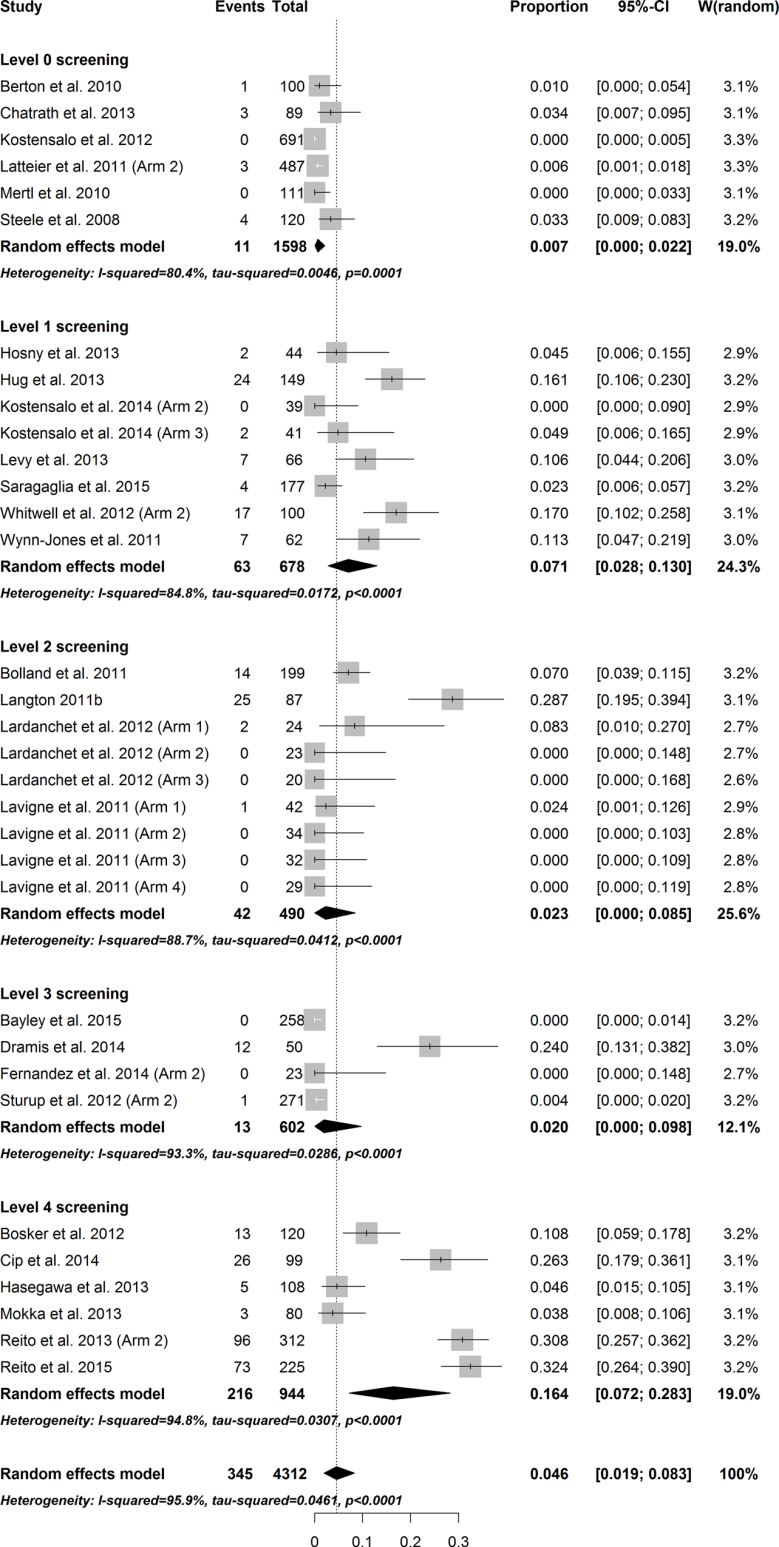
Forest plot of prevalence of ARMD in the LD THA group stratified by level of screening.

**Fig 4:**
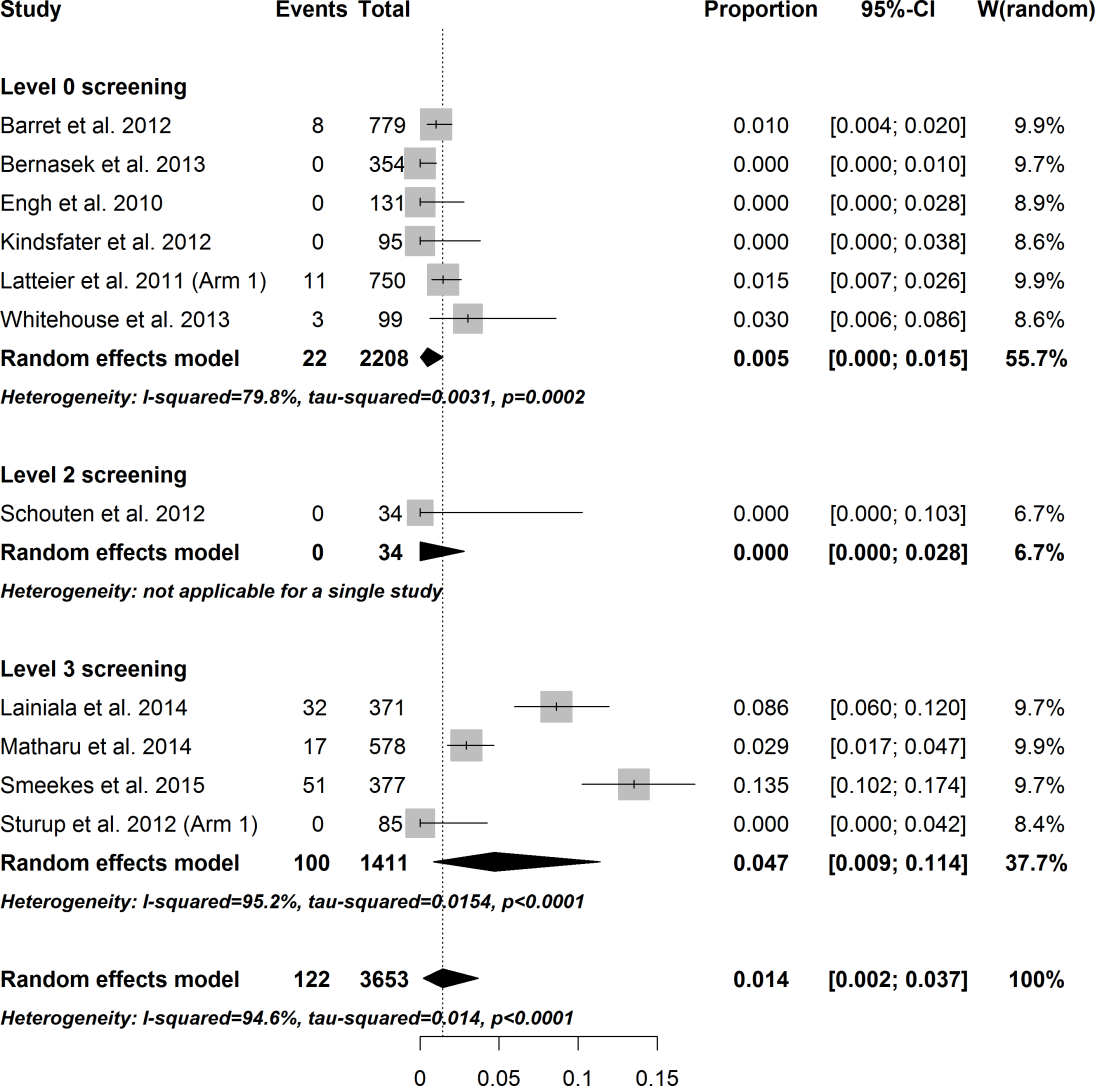
Forest plot of prevalence of ARMD in the MD THR group stratified by level of screening.

The total overall pooled estimate for the prevalence of revision confirmed ARMD among 145 study arms was 1.07% (CI: 0.69–1.49, I^2^ = 92.3%, p_heterogeneity_ < 0.001). In general, the amount of heterogeneity was high. Individual study arms were stratified according to the level of screening for ARMD and the implant concept. The individual weighted prevalences of ARMD in each study arm under the random effects model are shown in Figs 2–4. In the hip-resurfacing group, the overall pooled prevalence of ARMD was 0.43% (CI: 0.25–0.65). With more comprehensive screening, the higher pooled prevalence of ARMD was observed (Fig 2). In the LD THR group, the overall pooled prevalence of ARMD was 4.6% (CI: 1.94–8.32). Prevalence peaked in the study arms with Level 4 screening (Fig 3). The clear trend of higher prevalence of ARMD associated with an increased level of screening seen among resurfacings groups was not observed in this group. The overall pooled prevalence of revision confirmed ARMD in the MD THR group was 1.43% (CI: 0.21–3.70%). This group lacked study arms with Level 1 and Level 4 screening (Fig 4).

### Metaregression analysis

Metaregression was performed separately for hip resurfacings and LD THRs (Table 3). Metaregression was not performed in the MD THR group since no study arms were available for screening levels 1 and 4. For hip resurfacings, comprehensive screening (Level 4) was superior when compared with other levels, i.e., the prevalence of revision confirmed ARMD was significantly higher in level 4 studies when compared with others. An increase in follow-up time had a small, positive effect on the prevalence of ARMD. This association remained after adjusting for confounding variables. In the LD THR group, level 1 screening proved to be as good as level 4 screening. Screening levels 0.2 and 3 were inferior when compared with level 4 screening, i.e., the prevalence of ARMD was significantly lower in these levels compared with the level 4 study arms. These differences remained after adjusting for confounding variables.

**Table 3:** Results of the metaregression analysis in the HR and LD THR groups.

**Univariate regression analysis**		**Hip resurfacings**			**LD-THA**		
		β	SE	p-value	β	SE	p-value
**Screening**	Comprehensive (Level 4)	Reference	-	-	Reference	-	-
	Blood metal ions with targeted imaging (Level 3)	-0.117	0.051	0.022	-0.279	0.098	0.005
	Blood metal ions without imaging (Level 2)	-0.221	0.037	<0.001	-0.259	0.082	0.002
	Targeted Blood metal ions and/or imaging (Level 1)	-0.212	0.037	<0.001	-0.150	0.082	0.067
	No screening (Level 0)	-0.273	0.034	<0.001	-0.329	0.086	<0.001
**Multivariate regression analysis**		**Hip resurfacings**			**LD-THA**		
		β	SE	p-value	β	SE	p-value
**Screening**	Comprehensive (Level 4)	Reference	-	-	Reference	-	-
	Blood metal ions with targeted imaging (Level 3)	-0.137	0.051	0.007	-0.264	0.108	0.014
	Blood metal ions without imaging (Level 2)	-0.213	0.036	<0.001	-0.245	0.115	0.033
	Targeted CoCr and/or imaging (Level 1)	-0.220	0.037	<0.001	-0.141	0.090	0.12
	No screening (Level 0)	-0.261	0.034	<0.001	-0.320	0.117	0.006
**Mean FU**	Per one year	0.0054	0.003	0.045	-0.013	0.02	0.5
**Publication year**	Per one year	0.0034	0.003	0.3	-0.006	0.03	0.8

β = unstandardized regression coefficient, SE = standard error.

### Sensitivity analysis

**Fig 5:**
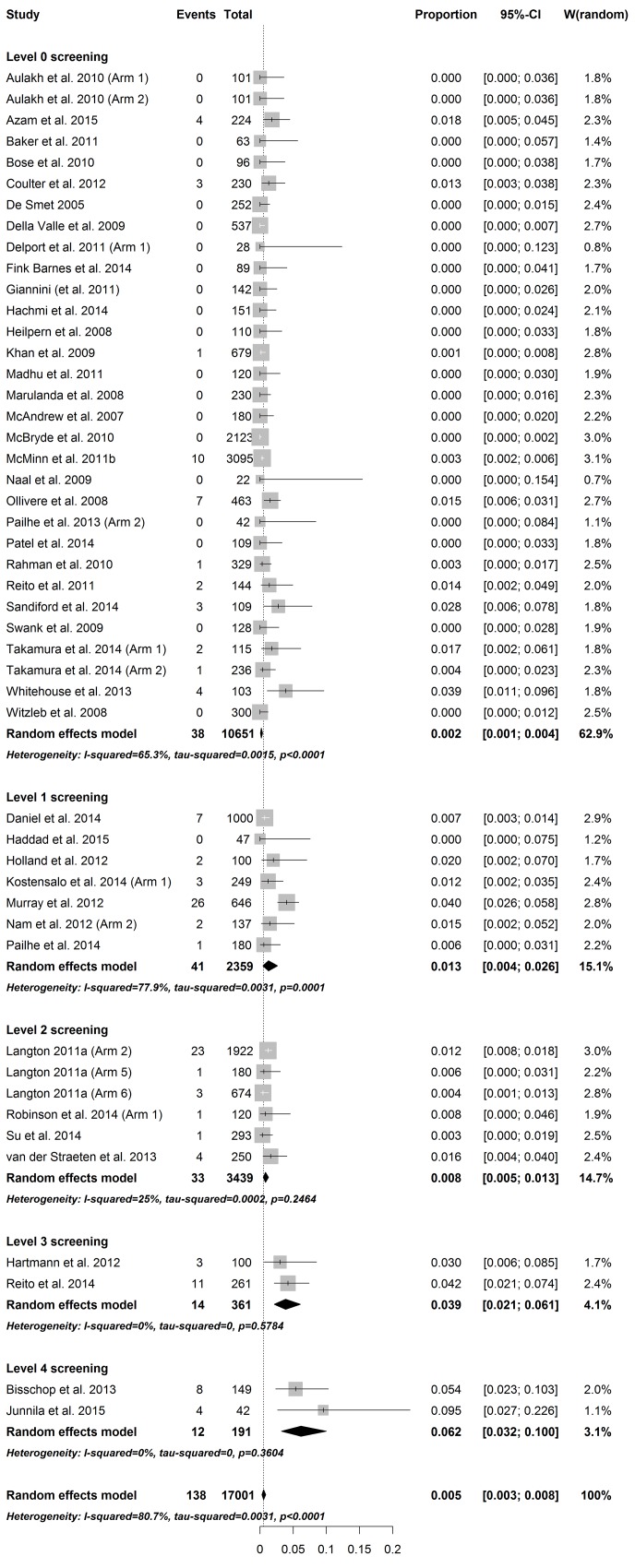
Forest plot of prevalence of ARMD in the BHR group stratified by level of screening.

All analyses were calculated using only study arms with Birmingham Hip Resurfacing, which has been the most used implant (48 arms). A trend was observed that showed an increased prevalence of ARMD associated with an increased level of screening (Fig 5). The results of the metaregression analyses were similar to those observed with all hip resurfacings with the exception of level 3, where no inferiority to level 4 screening was observed (Table 4). Screening levels 0,1 and 2 were significantly inferior to level 4.

**Table 4:** Results of the metaregression analysis in the BHR.

**Univariate regression analysis**		**Birmingham Hip Resurfacing**		
		β	SE	p-value
**Screening**	Comprehensive (Level 4)	Reference	-	-
	Blood metal ions with targeted imaging (Level 3)	-0.064	0.061	0.3
	Blood metal ions without imaging (Level 2)	-0.169	0.051	0.001
	Targeted CoCr and/or imaging (Level 1)	-0.141	0.051	0.006
	No screening (Level 0)	-0.212	0.048	<0.001
**Multivariate regression analysis**		**All study arms**		
		β	SE	p-value
**Screening**	Comprehensive (Level 4)	Reference	-	-
	Blood metal ions with targeted imaging (Level 3)	-0.64	0.90	0.2
	Blood metal ions without imaging (Level 2)	-2.06	0.75	0.001
	Targeted Blood metal ions and/or imaging (Level 1)	-1.51	0.74	0.003
	No screening (Level 0)	-1.94	0.67	<0.001
**Mean FU**	Per one year	0.012	0.068	0.2
**Publication year**	Per one year	0.14	0.10	0.2

β = unstandardized regression coefficient, SE = standard error.

## Discussion

Despite the marginal use of MoM hip replacements nowadays, the orthopedic community must bear the burden of a vast follow-up that has resulted from the widespread use of these devices over the past 15 years [130]. It is evident that patients with MoM hip replacements must be followed-up, at least clinically. However, there is paucity of information available regarding the optimal follow-up protocol and especially regarding the use of blood metal ion measurement and cross-sectional imaging. We must be rigorous and aim for the best possible and up-to-date evidence when constructing guidelines on how to manage patients with MoM replacements. Thus, we have performed a systematic literature review and meta-analysis to investigate the influence of the extent of the screening protocol on the prevalence of revision confirmed ARMD.

The overall pooled prevalence of confirmed prevalance ARMD was low. This is not a surprising finding considering that in most of the studies no screening was implemented other than conventional x-rays and clinical examination. The prevalence of ARMD was lowest in the study arms without screening (level 0). Moreover, these study arms also included the largest number of hips. Due to the weighting based on the sample sizes, the overall prevalence of ARMD does not, therefore, correctly highlight the current situation in patients with MoM hip replacements.

Heterogeneity between the studies was high. Firstly, there was a lot of variation in the implants used. There are many implant specific factors (clearance, hemispherity, carbon content, etc) that influence the wear of the bearing surface, and, therefore, bearing wear rates may differ greatly between different bearing systems [131,132]. Furthermore, both clinical studies and registry data show that there are major differences in the failure rates between different hip resurfacings and LD THRs [79,110,133]. These differences in failure rates are due to the modular taper-trunnion junction between the head and stem in the THRs, which is an additional source of metal debris due to corrosion and mechanical wear in the taper interface [134]. Secondly, when only study arms with the BHR implant were analyzed, high heterogeneity was still observed. The outcome variable assessed in our analysis was the revision rate for ARMD. Even if two studies implemented identical screening protocols, i.e., full coverage blood metal ion measurement and targeted cross-sectional imaging, very different prevalences of revision confirmed ARMD could still be observed. This is because indications for revision surgery can vary greatly between different surgeons and different hospitals. Some surgeons may prefer closer follow-up in cases where others would prefer revision surgery. The current literature lacks a specific definition for ARMD and especially the indications for revision. Due to these implant and inter-observer related differences, a high heterogeneity is observed.

Other confounding factors that may have influenced the observed prevalence of revision confirmed ARMD are the follow-up time and the publication year of the study. The prevalence of ARMD increases in a cumulative manner with increasing follow-up time [110]. ARMD may manifest as early as two years postoperatively and a late occurrence after ten-years of follow-up is also possible [2,15]. Thus, we included follow-up time as a confounding variable in our metaregression analysis to investigate whether prevalence of ARMD is a matter of long enough follow-up time or a matter of the screening protocol used. The follow-up time had no influence on the observed prevalence of ARMD. Therefore, our results suggests that even if the prevalence of revision confirmed ARMD would increase with increasing follow-up time, this association would not be observed due to the stronger effect of the screening protocol used.

The year of publication was also an important variable to consider as a confounder. We believe that there has been considerable publication bias in the MoM literature during recent years. As a result, there has been a strong tendency to publish as high as possible prevalences of pseudotumours and ARMD. Several extremely poorly functioning MoM hip replacements in have been in widespread use, and during the last two years numerous studies have been published that report the outcome of these poorly functioning implants. In most of the studies, the primary aim has been to elucidate the higher than anticipated failure rate due to ARMD. The “higher than anticipated” failure rate reflects the actual situation that we are facing nowadays with several MoM hip replacements, but from the perspective of the literature review we are facing publication bias. We do not have a sufficient number of studies where novel screening methods have not been used (level 0) and that report the results of the use of MoM hip replacements before the problems with MoM bearings surfaces became evident and blood metal ion measurements and cross-sectional imaging became popular. Moreover, prior to 2010, MoM hip replacements were popular and there was a trend towards positive results instead. The trend towards positive results can be observed in the numerous studies that report favorable results with the BHR device. Furthermore, to support this statement, one can see that there are no studies prior to 2010 that have reported, for example, the results of the ASR XL THR, which was eventually shown to have been disastrous [79,110,117]. As was the case with follow-up time, we did not observe any influence of the year of publication on the observed prevalence of ARMD. Therefore, our results reliably highlight the important role that the screening protocol has in influencing the prevalence of ARMD.

In the hip-resurfacing group, metaregression analysis suggested that level 4 screening was superior to all other screening levels and especially when compared to level 3. We consider this to be a novel finding. The main difference between these screening protocols is that when changing from level 3 to level 4, we ought to refer many patients with non-elevated metal ion levels or without complaints for cross-sectional imaging since these patients are not imaged in level 3 screening. We observed slightly higher pooled prevalence of revision confirmed ARMD in study arms with level 4 screening compared with study arms with level 3 screening. This difference in pooled estimate for the prevalence of ARMD is one benefit of screening patients without relevant clinical findings.

Our results suggest that this movement from targeted imaging to full coverage imaging is useful with regard to the prevalence of revision confirmed ARMD. However, the economical aspect and cost-effectiveness of this “transition” should be carefully assessed. When only BHR implants were analyzed, level 3 screening was not found to be inferior to level 4 screening. This would indicate that full coverage imaging would not be beneficial in patients with BHR. However, in this subgroup the analysis might be underpowered. These results should be kept in mind especially when the economics of the surveillance of patients with MoM hip replacements are considered since cross-sectional imaging is the most expensive procedure in the screening process.

In contrast to imaging, especially MRI, metal ion measurement is a readily available, inexpensive screening modality that should be used in the surveillance of patients with MoM hip replacements [15]. Current MHRA guidelines do not give instructions on how to perform metal ion measurement in asymptomatic patients with MoM hip resurfacing [135]. In our institution, however, metal ion measurement is a routine follow-up measure in all hip-resurfacing patients. As our previous study suggests, the measurement of metal ions is beneficial in patients without complaints since ARMD is often seen in asymptomatic patients [15]. The results of our current study also imply the usefulness of routine metal ion measurement. A comparision of the non-routine metal ion measurement (level 1) with the routine measurement (level 3) would have been more sensible from this point of view, but for the sake of simplicity we used level 4 as our reference in our metaregression analysis. It should be noted, however, that confidence intervals for pooled prevalences of revision confirmed ARMD in the level 1 and level 2 screening study arms barely overlap with those seen in the level 3 study arms. Moreover, a distinct change in the prevalence of ARMD is seen when moving from screening levels 1–2 to screening levels 3–4. To conclude, our results suggest that routine metal ion measurement is useful in patients with hip resurfacing or BHR. More importantly, routine metal ion measurement should be performed along with targeted or full coverage imaging.

Results in the LD THR group were different than those in the hip-resurfacing group. Surprisingly, level 1 screening was equal to level 4 screening. Moreover, the pooled prevalence of ARMD with level 2 and 3 screening was clearly smaller than in study arms with level 1 screening. This is probably a biased result due to sample sizes since most study arms in level 2 screening included less than 40 hips, and these numbers might be too small to detect the actual failure rate. However, in study arms with level 3 screening there were two cohorts with more than 250 hips, and surprisingly a small prevalence of ARMD was observed in these study arms. For example, Bayley et al. had no revision due to ARMD after extensive screening [100].

The major issue with LD THRs is taper corrosion that may possibly release more toxic wear debris than that originating from the bearing surfaces [134]. Most probably, severe ARMD may be observed even in the presence of non-elevated (< 5 ppb) metal ion levels as a result. This would also explain why the clearly highest prevalence of ARMD was seen in the study arms with level 4 screening, i.e., in those studies where all patients were screened with cross-sectional imaging independent of blood metal ion levels. Hence, in patients with LD THRs, a low threshold for imaging is recommended even in the presence of normal metal ion levels.

## Conclusions

The aims of this study were successfully achieved. Based on our systematic literature review and metaregression analysis, the overall pooled prevalence of revision confirmed ARMD represented in the current MoM literature is low. However, this seems to be a consequence of the use of the conventional follow-up protocol, namely x-rays and clinical examination, in the majority of the published studies. The implementation of the novel screening protocol results in a clearly higher prevalence of ARMD. The highest prevalence of revision confirmed ARMD was seen when all patients had undergone both blood metal ion measurement and cross-sectional imaging. These outcomes were irrespective of the follow-up time or study publication year. With regard to hip resurfacings, routine cross-sectional imaging regardless of clinical findings is advisable. Moreover, targeted metal ion measurement and/or imaging are not sufficient in the screening for ARMD in any implant concept. However, economical aspects should be considered when choosing the preferred screening level.

## Supporting Information

S1 Checklist PRISMA 2009 checklist. (DOC)

S1 Datafile (XLSX)
